# Suicidal behaviour after first-episode psychosis: results from a 1-year longitudinal study in Portugal

**DOI:** 10.1186/s12991-021-00356-0

**Published:** 2021-07-06

**Authors:** Ricardo Coentre, Alexandra Fonseca, Tiago Mendes, Ana Rebelo, Elisabete Fernandes, Pedro Levy, Carlos Góis, Maria Luísa Figueira

**Affiliations:** 1 Department of Psychiatry, Centro Hospitalar Universitário Lisboa Norte, Av Prof. Egas Moniz, 1649-035 Lisboa, Portugal; 2 Psychiatric University Clinic, Faculdade de Medicina, Universidade de Lisboa, 1649-028 Lisboa, Portugal; 3 Biomathematics Laboratory, Faculdade de Medicina, Universidade de Lisboa, 1649-028 Lisboa, Portugal

**Keywords:** Suicidal behaviour, First-episode psychosis, Depression, Schizophrenia

## Abstract

**Background:**

Suicide is one of the main causes of excess of premature death in psychotic patients. Published studies found that suicide risk begins in ultra-high risk of psychosis and continues in early years of the disease. Previous studies identifying predictive and risk factors associated with suicidality in first-episode psychosis (FEP) are highly inconsistent. Also, there are relatively few longitudinal studies on suicidal behaviour in FEP. The aim of this study was to examine prevalence, evolution and predictors of suicidal behaviour at baseline and the 12-month follow-up in patients presenting with FEP.

**Methods:**

One hundred and eighteen patients presenting with FEP were recruited from two early psychosis units in Portugal. A comprehensive assessment examining socio-demographic and clinical characteristics was administered at baseline and the 12-month follow-up. Odds ratio were calculated using logistic regression analyses. McNemar test was used to evaluate the evolution of suicidal behaviour and depression prevalence from baseline to 12 months of follow-up.

**Results:**

Follow-up data were available for 60 participants from the 118 recruited. Approximately 25.4% of the patients had suicidal behaviour at the baseline evaluation, with a significant reduction during the follow-up period to 13.3% (*p* = 0.035). A multivariate binary logistic regression showed that a history of suicidal behaviour and depression at baseline independently predicted suicidal behaviour at baseline, and a history of suicidal behaviour and low levels of total cholesterol predicted suicidal behaviour at the 12-month follow-up. A significant proportion of patients also had depression at the baseline evaluation (43.3%), with the last month of suicidal behaviour at baseline independently predicting depression at this time.

**Conclusions:**

The findings of our study indicate that suicidal behaviour was prevalent on the year after FEP. Patients with a history of suicidal behaviour, depression at baseline and low levels of cholesterol should undergo close evaluation, monitoring and possible intervention in order to reduce suicide risk in the early phases of psychosis.

**Supplementary Information:**

The online version contains supplementary material available at 10.1186/s12991-021-00356-0.

## Background

Suicide and cardiovascular disorders are the two main causes of excess or premature death in psychotic patients [1]. About 5% of patients with schizophrenia die by suicide, and about 50% have suicidal ideation or attempt suicide [2, 3]. Suicide risk differs across the various stages of the disorder, with the highest rates occurring in the early phases [4]. Recent studies found that this suicide risk begins at ultra-high risk of psychosis (UHR) stage and continues in early years of the disease [5, 6]. Patients experiencing first-episode psychosis (FEP) have a 60% increased risk of suicide in the first year of treatment compared with those in subsequent phases [7]. The prevalence of suicidal ideation, a consistent antecedent of suicidal behaviour (attempts and completed suicide), ranges from 26.2% to 56.5% during the initial presentation of FEP [8, 9]. The prevalence of suicide attempts in the years following FEP ranges from 2.9% to 18.2% and suicide from 0.4% to 4.29% [10].

Therefore, the first years of psychotic disorders represent an important challenge for Public Health and Psychiatry. Consequently, researchers have sought to identify predictive and risk factors associated with suicidality in FEP; however, the findings are highly inconsistent. Younger age of onset of psychosis [8, 11, 12] and drug and alcohol use [13–15] are factors associated with suicidality in FEP. As in the general population, previous suicidal behaviour, e.g. suicide attempts, has been identified as one of the most significant predictors of suicidal risk in FEP [16–18]. However, while results are mixed regarding gender [8, 11, 19–24], duration of untreated psychosis [7, 8, 22], positive psychotic symptoms [7, 25, 26] and insight [8, 15, 24, 27–29], treatment compliance has been demonstrated to reduce suicide risk [30].

Current or past significant depressive symptoms are associated with suicide risk in patients after FEP [15, 31–34]. The prevalence of depressive symptoms after FEP ranges from 14.15% to 44.80%. Studies evidenced that depressive symptoms diminished with follow-up time and treatment, but remained significant even 10 years after FEP [10, 35]. Previous studies showed that the most depressive symptoms occur either in prodrome [36] or during FEP [37, 38]. Rarely do depressive symptoms occur de novo in recovery following FEP [31]. Usually, depressive symptoms follow the course of positive psychotic symptoms and remit with antipsychotic treatment [39], although there are some patients with psychosis who maintain persistent depressive symptoms beyond the acute phase and do not respond to antipsychotic treatment. Co-morbid depression and psychosis increase the risk of suicidality and relapse [40, 41]. It has been suggested that early identification and intervention for depression in the early phases of psychosis may constitute an important strategy in the prevention of suicide [4, 42].

Published studies suggest that early intervention services—including short treatment delays, maintaining adherence to therapy and specialised programmes—reduce the risk of suicide [43, 44], which is an important aim of early intervention teams. Despite the relevance of this theme, there are relatively few longitudinal studies on suicidal behaviour in FEP and early phases of psychosis. Additionally, published studies have some methodological limitations that we tried to overcome. First, many published studies only included first-episode schizophrenia patients [12, 29, 45]. It is known that a diagnosis of FEP is unstable, with the likelihood of subsequent changes in the first years of the illness [46]. Therefore, it is suggested that studying a broader diagnostic sample of FEP with affective and non-affective psychosis would be more accurate in estimating suicidal behaviour. Second, some studies included heterogeneous samples that mixed patients at different stages of their illness, including chronic and early psychosis patients. Third, many past studies only included hospitalised patients, excluding milder forms of psychotic disorders. Fourth, most previous studies used retrospective or cross-sectional designs, which are characterised by inherent difficulties in establishing predictive factors.

Greater understanding and knowledge around suicide risk profiles and predictors of suicide in psychotic patients will enable the development of preventive strategies. Appropriate monitoring and managing of suicide risk will be important for services working with early psychosis populations. Prospective studies in patients with FEP are necessary to better identify predictive and risk factors for suicidal behaviour in early psychosis. The prevalence and methods of suicidal behaviour differ between countries and cultures. The literature indicates that socio-cultural factors and healthcare resources may condition the risk of suicide [47–49]. Therefore, it is important to have studies from different countries and cultures.

The main aims of this study were: (1) to examine the prevalence and evolution of suicidal behaviour soon after FEP (baseline) and 12 months of follow-up and (2) to determine baseline predictors of suicidal behaviour soon after FEP and at the 12-month follow-up. We also examined the prevalence of depression, its evolution in the 12 months following FEP and the predictors of depression at baseline and follow-up. We hypothesised that affective psychosis, previous suicidal behaviour and depressive symptoms at baseline would predict the occurrence of suicidal behaviour at baseline and the 12-month follow-up.

## Methods

### Participants and setting

The study was carried out at two specialist psychiatric services within Portuguese hospitals: Hospital Vila Franca de Xira (in Vila Franca de Xira with a catchment area of 245.000 individuals) and Centro Hospitalar Universitário Lisboa Norte (in Lisbon with a catchment area of 350.000 individuals). The selected patients were from two early intervention teams: the First-Episode Psychosis Program (PPEP) at Hospital Vila Franca de Xira (Vila Franca de Xira) and Programa de Intervenção nas Fases Iniciais da Psicose (PROFIP) at Centro Hospitalar Universitário Lisboa Norte (Lisbon). Both programmes are comprehensive, specialised mental health services for FEP patients originating from geographically defined catchment areas in Vila Franca de Xira and north region of Lisbon, Portugal. Vila Franca de Xira Hospital is a secondary care general hospital in the north metropolitan area of Lisbon. Centro Hospitalar Universitário Lisboa Norte is a tertiary care general university hospital, which interacts closely with the Faculty of Medicine, University of Lisbon. The populations served by the two hospitals live mainly in the suburban and urban areas, respectively, of the metropolitan region of Lisbon. A description of the PROFIP programme was published elsewhere [50].

All FEP patients underwent an assessment by trained personnel as soon as possible after contact with the team. The mean length from service entry to baseline assessment was 5.7 days (SD: 1.8). The programmes consisted of low-dose second-generation antipsychotic medication and individual and group psychosocial treatment, namely family intervention.

The participants met the following inclusion criteria at baseline: (1) age between 16 and 40 years; (2) DSM-IV (American Psychiatric Association, 1994) diagnosis of a psychotic disorder (schizophrenia, schizophreniform disorder, schizoaffective disorder, delusional disorder, bipolar psychotic disorder, major depressive disorder with psychotic features, brief psychotic disorder, cannabis-induced psychosis and psychosis not otherwise specified); (3) living in the geographical catchment of the PPEP or PROFIP services; (4) adequate comprehension of Portuguese language; (5) experiencing FEP with less than 6 months of antipsychotic medication. Exclusion criteria at baseline included: (1) head injury, neurological illness, or any other medical condition presenting with psychiatric symptoms (2) history of past full-blown psychotic episodes, either non-affective or affective, as defined in the Diagnostic and Statistical Manual of Mental Disorders, IV Edition (American Psychiatric Association, 1994); (3) inability to understand and complete the assessments.

The study included all eligible consecutive patients who met the study criteria for the period January 2017 to April 2020. The patients were evaluated at two time points: a baseline evaluation after the clinical stabilisation of FEP; 12 months after the baseline assessment. Follow-up data were available for 60 participants from the 118 recruited. The study complied with the ethical principles of good practice embodied in the Declaration of Helsinki. Responsible ethical committees within both hospitals approved the study, and all participants provided informed consent.

### Clinical assessments

The clinical assessment included evaluations regarding socio-demographics and clinical data, including age, marital status, education, occupational status, living area (urban or rural), medical history, education, family history of mental illness, presence of stressful life events occurring in the past year before entering the study, mode of onset of FEP, substance use and type and dose of antipsychotic medication. The baseline and 12-month follow-up diagnoses were obtained using all available information, including informant history and medical records. The Operational Criteria Checklist for Psychotic Illness and Affective Illness (OPCRIT +) instrument was used to obtain the diagnosis [51, 52]. The checklist ratings were entered into the OPCRIT + software, which generates a diagnosis for the main categories of affective and psychotic disorders, as defined by the DSM-IV, which is the major classification system. The duration of untreated psychosis (DUP) was measured using the Nottingham Onset Schedule (NOS) [53]. DUP was defined as the period of time between the onset of psychotic symptoms and the initiation of treatment with antipsychotic medication. Symptom levels were evaluated using means of the three subscales used to evaluate positive symptoms, negative symptoms and the general psychopathology of the Positive and Negative Syndrome Scale (PANSS) [54]. Suicidal behaviour was assessed at baseline and follow-up evaluations using an instrument developed by Melle [18], which consists in three questions about the last month and lifetime suicidal thoughts, plans or attempts.

The information was cross-checked with the medical records. “Suicidal thoughts” were considered a preoccupation or recurrent thoughts of suicide without a specific plan. “Suicide plans” were considered as the presence of a specific plan to suicide. “Suicide attempts” were defined as a self-non-fatal act with a suicidal intent. The most severe form of suicidal behaviour reported for a lifetime or the past month was registered.

The baseline and 12-month follow-up assessments of depressive symptoms were conducted using the Beck Depression Inventory (BDI). The BDI is a widely used self-report questionnaire designed to measure the severity of depression in individuals between the ages of 13 and 80 years. It contains 21 items, which are rated on a four-point scale according to how the patients felt in the previous two weeks. The scores obtained for the single item are summed to provide a single total score. We used 14 as the global cut-off score to determine patients’ depression (depression ≥ 14). We used the Portuguese version, which was translated into Portuguese and adapted to the Portuguese population [55–57].

For the alcohol screening, the Michigan Alcohol Screening Test (MAST) was used. This is a 22-item self-questionnaire requiring yes/no answers, with one point each. A total score of 6 or more indicates hazardous alcohol consumption or alcohol dependence. The Portuguese-translated version, which was adapted to the Portuguese population, has been used before [58, 59]. Functioning was measured using the Global Assessment of Functioning Scale (GAF) [60]. Adherence to medication was evaluated by Medication Adherence Rating Scale (MARS). The MARS is a ten-item self-report measure of medication adherence in psychosis [61]. The total score ranges from 0 to 10 with a higher score indicating better adherence. We used the translated and validated scale to the Portuguese population [62].

### Statistical analysis

Statistical analyses were conducted using the Statistical Package for Social Science (SPSS), version 26 (Inc, 2020). Due to non-normality in all the explored statistics, non-parametric tests were used. In the between-group comparisons, categorical variables were examined using Chi-square or Fisher’s exact test (if 20% of expected frequency was ≤ 5 or any expected frequency was < 1) and continuous variables with the Mann–Whitney U test.

The primary analysis of this study focused on identifying factors predicting the occurrence of suicidal behaviour at baseline and 12 months after the occurrence of FEP. We also evaluated the predictors of depression in FEP patients (at baseline and 12 months after FEP). Suicidal ideation/behaviour was collapsed into a binary variable suicidal/non-suicidal (i.e. presence/absence of suicidal thoughts, plans or attempts). We also considered depressive/not depressive as well as the above-mentioned cut-off on the BDI scale. First, a univariate binary logistic regression was conducted, with suicidal behaviour as the dependent variable and demographic and clinical variables as the candidate predictors. Second, variables with a significant *p* value in the preceding analysis were then entered into a multivariate binary regression model to determine which factors independently predicted suicidal behaviour at baseline and the 12-month follow-up. A stepwise method was used with a forward selection of predictors. The same procedure was conducted, with depression as the dependent variable. Collinearity evaluations were performed by examining correlation matrices for all the variables, tolerance and variance inflation factors.

We used the McNemar test to evaluate the evolution of suicidal behaviour and depression and compare the results at baseline with those at 12 months. The Wilcoxon test for repeated measures was used to evaluate the evolution of depression from the BDI mean score. The level of statistical significance was set at *p* < 0.05.

## Results

### Characteristics of the sample

The sample comprised 118 patients consecutively admitted to PPEP (*n* = 39) and PROFIP (*n* = 79). There were no differences between the two groups in terms of sex and employment. The patients from the PROFIP team were younger (mean years 24.2 vs. 29.61; *p* = 0.003) and of a higher educational level (mean years of education 11.7 vs. 10.1; *p* = 0.016) (Mann–Whitney U-test for both). Of the 118 participants included in the study, 76.3% (*n* = 90) were male. The mean age of the sample at intake was 26.1 years (SD = 7.1). The majority (49.2%) of the patients were diagnosed with schizophrenia spectrum disorders (schizophrenia: *n* = 48; delusional disorder: *n* = 8; schizoaffective disorder: *n* = 2). Thirty-nine (33%) were diagnosed with other psychosis diagnosis, with 8 patients with a brief psychotic disorder, 23 with a non-specified diagnosis of psychosis and 8 with a cannabis-induced psychosis diagnosis. Twenty-one (17.8%) participants had affective psychosis (*n* = 8 had bipolar affective disorder with psychotic features, and *n* = 13 had major depression with psychotic features). Table 1 shows the socio-demographic and clinical baseline variables of the sample.

**Table 1:** Socio-demographic and clinical baseline variables of the sample

Variables	First-episode psychosis patients *n* = 118
Age years—mean (SD)	26.1 (7.10)
Gender—n (%)	
Female	28 (23.7%)
Male	90 (76.3%)
Education years—mean (SD)	11.24 (3.2)
Unemployment—n (%)	51 (43.2%)
Marital status—n (%)	
Living with partner/married	14 (11.9%)
Single/divorced	104 (88.1%)
Hospitalisation baseline—n (%)	
Yes	101 (85.6%)
No	17 (14.4%)
Cannabis use—n (%)	
Yes	75 (63.6%)
No	43 (36.4%)
Psychiatric family history—n (%)	76 (64.4%)
DUP – days, median (SD)	84 (643.3)
Diagnoses—n (%)	
Schizophrenia spectrum diagnosis	58 (49.2%)
Affective psychosis spectrum diagnosis	21 (17.8%)
Other psychosis diagnosis	39 (33%)
PANSS score—mean (SD)	
PANSS positive subscale	21.24 (8.09)
PANSS negative subscale	15.86 (7.41)
PANSS general subscale	35.97 (8.80)
GAF—mean (SD)	43.40 (18.28)
BDI—mean (SD)	13.18 (10.10)
MARS—mean (SD)	6.08 (2.26)
Total Cholesterol mg/dL—mean (SD)	153.43 (31.72)

*SD* standard deviation, *DUP* duration of untreated psychosis, *PANSS* Positive and Negative Syndrome Scale, *GAF* Global Assessment of Functioning, *BDI* Beck Depression Inventory, *MARS* Medication Adherence Rating Scale

Schizophrenia-spectrum disorder included schizophrenia, schizophreniform disorder, delusional disorder and schizoaffective disorder. Affective psychosis included bipolar disorder with psychotic symptoms and depressive disorder with psychotic symptoms. Other psychosis included acute and transient psychotic disorders, brief psychotic disorder, cannabis-induced psychosis and psychosis not otherwise specified

Of the initial cohort of 118 patients who completed the baseline assessment, 60 completed the follow-up assessment. We lost contact with 58 patients for the following reasons: 30 moved to other catchment areas in Portugal; 15 moved to other countries and 13 could not be contacted for the follow-up. Non-completers and completers groups had no significant differences regarding socio-demographics and clinical variables, with the exception of more males (*p* = 0.009) and lower affective psychosis diagnoses (*p* = 0.030) on the non-completers group.

Comparisons of socio-demographics and clinical characteristics between the affective psychosis and schizophrenia spectrum disorder diagnoses are shown in Additional file 1: Table S1.

### Prevalence of suicidal behaviour

Forty-two (35.6%) participants had a history of suicidal behaviour prior to entering the study, with 34.7% (*n* = 41) of the cohort having suicidal ideation, 17.80% (*n* = 21) suicidal plans and 9.32% (*n* = 11) a suicide attempt. Overdose of medication was the most frequently used attempted suicide method (54.5%)), followed by jumping from a height (18.2%), strangulation (18.2%) and phlebotomy (9.1%).

For the total initial FEP group, suicidal behaviour in the last month was found for 25.4% (*n* = 30) of the cohort, with 15.3% of them experiencing suicidal ideation, 4.2% suicidal plans and 5.9% suicide attempts. The most frequently used methods of attempted suicide in the last month were overdose of medication, jumping from a height and hanging (1.7% in each group). One patient attempted suicide through phlebotomy.

For the participants who completed the 12-month evaluation, 13.3% (*n* = 8) had suicidal behaviour, 8.3% suicidal ideation, 3.3% suicidal plan and 1.7% a suicide attempt. The only suicide attempt was overdose of medication.

Twelve patients (40%) who reported suicidal behaviour at baseline evaluation had no history of suicidal behaviour. Only one patient that reported suicidal behaviour at follow-up evaluation had no history of suicidal behaviour.

With regard to the longitudinal evolution of suicidal behaviour, a statistically significant decrease at the 12-month follow-up was found (25.4% vs. 13.3%; *p* = 0.035).

### Univariate associations with suicidal behaviour

The associations of suicidal behaviour at baseline and 12 months of follow-up with the demographic and clinical factors are shown in Table 2. Patients with suicidal behaviour at baseline were significantly more likely to have a history of suicidal behaviour, baseline affective psychosis diagnosis and baseline depression. Suicidal behaviour patients at 12 months of follow-up were found to have a significantly greater history of suicidal behaviour at baseline, baseline suicidal behaviour in the last month and lower total cholesterol at baseline.

**Table 2:** Demographic, pre-treatment and baseline predictors of suicidal behaviour

Variables of interest	Baseline suicidal behaviour				12-month suicidal behaviour			
	Patients with suicidal behaviour (*n* = 30)	Patients without suicidal behaviour (*n* = 88)	OR (95% CI)	*p* value	Patients with suicidal behaviour (*n* = 8)	Patients without suicidal behaviour (*n* = 52)	OR (95% CI)	*p* value
Socio-demographics								
Male/female sex, n (%)	21(70.0)/9(30.0)	69(78.4)/19(21.6)	1.56 (0.61–3.95)	0.352	4 (50.0)/4 (50.0)	36/16	2.25 (0.50–10.14)	0.291
Age at entry, mean (SD)	26.83 (7.49)	25 (7.01)	0.98 (0.93–1.04)	0.504	30.13 (9.91)	26.63 (7.60)	0.95 (0.87–1.04)	0.252
Years of education, mean (SD)	11.10 (3.60)	11.28 (3.01)	1.02 (0.89–1.16)	0.786	11.88 (3.56)	11.19 (3.33)	0.94 (0.75–1.18)	0.588
Married/with partner, n (%)	5 (16.7)	9 (10.2)	1.76 (0.54–5.73)	0.351	1 (12.5)	7 (13.46)	1.34 (0.14–13.25)	0.801
Living alone, n (%)	1 (3.3)	11 (12.5)	0.24 (0.03–1.95)	0.183	0 (0)	8 (15.38)	0.000	0.999
Employed/student, n (%)	16 (53.3)	51 (57.9)	0.83 (0.36–1.91)	0.659	6 (75.0)	2 (3.85)	2.20 (0.41–11.95)	0.361
Pre-treatment illness characteristics								
Family history of mental disorder, n (%)	20 (66.67)	56 (63.6)	1.14 (0.48–2.74)	0.765	7 (87.5)	1 (1.92)	3.11 (0.35–27.43)	0.307
History of suicidal behaviour, n (%)	18 (60.0)	24 (27.3)	4.00 (1.68–9.53)	0.002	7 (87.5)	1 (1.92)	13.22 (1.51–116.01)	0.020
History of substance use, n (%)	20 (66.67)	55 (62.5)	1.20 (0.50–2.87)	0.682	6 (75.0)	2 (3.85)	2.03 (0.37–11.05)	0.412
History of alcohol abuse, n (%)	15 (50.0)	37 (42.0)	1.38 (0.60–3.17)	0.449	3 (37.5)	5 (9.62)	0.89 (0.19–4.11)	0.877
Baseline clinical characteristics								
DUP, median (SD) days	28.00 (984.61)	92.00 (473.95)	1.00 (0.99–1.00)	0.204	19 (114.55)	98 (770.42)	1.00 (0.99–1.01)	0.291
Hospitalisation, n (%)	27 (90.0)	74 (55.7)	1.70 (0.45–6.40)	0.430	0 (0.0)	8 (15.38)	0.000	0.999
Tobacco use, n (%)	18 (60.0)	49 (55.7)	1.19 (0.51–2.77)	0.680	6 (75.0)	2 (3.85)	2.57 (0.47–13.94)	0.274
Last-month baseline suicidal behaviour	–	–	–	–	5 (62.5)	3 (5.77)	5.56 (1.16–26.70)	0.032
Diagnostic categories								
Schizophrenia-spectrum disorder, n (%)	11 (36.67)	47 (53.4)	0.51 (0.21–1.18)	0.116	2 (25.0)	6 (11.54)	0.29 (0.05–1.55)	0.146
Affective psychosis, n (%)	9 (30.0)	12 (12.6)	2.71 (1.01–7.31)	0.048	4 (50.0)	4 (7.69)	3.33 (0.72–15.37)	1.123
Other psychosis, n (%)	8 (26.7)	29 (32.9)	0.74 (0.29–1.86)	0.522	2 (25.0)	6 (11.54)	1.11 (0.19–6.24)	0.905
Symptom severity and functioning								
PANSS positive symptoms, mean (SD)	20.67 (8.05)	21.43 (8.14)	1.01 (0.96–1.07)	0.653	20.63 (7.84)	18.85 (7.29)	0.97 (0.88–1.07)	0.521
PANSS negative symptoms, mean (SD)	17.53 (8.48)	15.30 (6.98)	0.96 (0.91–1.01)	0.155	17.88 (6.643)	17.31 (7.237)	0.99 (0.89–1.09)	0.832
PANSS general symptoms, mean (SD)	39.07 (9.81)	34.91 (8.22)	0.95 (0.90–0.99)	0.031	39.63 (6.02)	35.69 (9.34)	0.96 (0.89–1.03)	0.260
BDI, mean (SD)	18.47 (11.434)	11.42 (8.93)	0.93 (0.89–0.97)	0.002	20.50 (12.11)	12.48 (9.87)	0.93 (0.87–1.00)	0.054
Depression, n (%)	20 (66.7)	32 (36.4)	3.50 (1.46–8.39)	0.005	6 (75.0)	2 (3.85)	4.80 (0.88–26.14)	0.070
GAF, mean (SD)	44.17 (17.52)	43.14 (18,62)	0.99 (0.97–1.02)	0.789	43.75 (15.53)	48.00 (17.60)	1.01 (0.97–1.06)	0.516
MARS, mean (SD)	5.50 (1.78)	6.23 (2.39)	1.15 (0.96–1.39)	0.130	5.75 (2.66)	5.79 (2.30)	0.97 (0.92–1.03)	0.304
Laboratory								
Total cholesterol	154.50 (32.56)	153.07 (31.62)	0.999 (0.986–1.012)	0.830	170.88 (26.2)	146.17 (30.518)	0.97 (0.95–0.99)	0.045

*DUP*  duration of untreated psychosis, *PANSS*  Positive and Negative Syndrome Scale, *BDI*  Beck Depression Inventory, *GAF*  Global Assessment of Functioning, *MARS*  Medication Adherence Rating Scale, *SD*  standard deviation

Schizophrenia-spectrum disorder included schizophrenia, schizophreniform disorder, delusional disorder and schizoaffective disorder

Affective psychosis included bipolar disorder with psychotic symptoms and depressive disorder with psychotic symptoms

Other psychosis included acute and transient psychotic disorders, brief psychotic disorder, cannabis-induced psychosis and psychosis not otherwise specified

### Predictors of suicidal behaviour in the multivariate model

A multivariate binary logistic regression analysis showed that a history of suicidal behaviour and depression at baseline predicted suicidal behaviour at baseline. A history of suicidal behaviour and low baseline total cholesterol predicted suicidal behaviour at 12 months of follow-up (Table 3).

**Table 3:** Multivariate logistic regression analysis for predictors of baseline and 12-month suicidal behaviour in first-episode psychosis patients^a,b^

Variables in the equation	B	SE	Wald	df	*p* value	OR	95% CI
Baseline							
Depression baseline	1.087	0.463	5.514	1	0.019	2.96	1.20–7.34
History of suicide behaviour	1.237	0.456	7.343	1	0.007	3.44	1.41–8.43
Constant	− 0.153	0.367	0.175	1	0.676	0.86	
Final model: Nagelkerke R^2^ = 0.185, χ^2^ = 15.850, p < 0.0001 Hosmer and Lemeshow test supported the goodness of fit of the model (χ^2^ = 0.006, df = 2, p = 0.997)							
12-month follow-up							
History of suicide behaviour	2.849	1.172	5.910	1	0.015	17.27	1.74–171.65
Total cholesterol baseline	− 0.030	0.014	4.411	1	0.036	0.97	0.94–0.99
Constant	5.643	2.369	5.674	1	0.017	282.31	
Final model: Nagelkerke R^2^ = 0.373, χ^2^ = 13.600, p = 0.001 Hosmer and Lemeshow test supported the goodness of fit of the model (χ^2^ = 12.657, df = 8, p = 0.124)							

^a^Affective psychosis diagnosis and PANSS general were entered into stepwise logistic regression model, were excluded as predictors of baseline suicidal behaviour

^b^Last month baseline suicidal behaviour were entered into stepwise logistic regression, were excluded as predictors of 12-month suicidal behaviour

### Prevalence and predictors of depression

The longitudinal evolution of depression prevalence showed a significant decrease between baseline and the 12-month follow-up (43.3% vs. 20.0%; *p* = 0.014).

A univariate analysis of predictors of baseline depression revealed that depressed patients had a significantly higher history of suicidal behavioural (OR: 2.29; *p* = 0.035). Also, depressed patients at baseline had a higher prevalence of suicidal behaviour in the last month (OR: 3.50; *p* = 0.005), which was the only variable that maintained a statistical significance in the multivariate analysis (OR: 2.94; 95% IC: 1.19–7.27; *p* = 0.020). None of socio-demographic or clinical baseline factors analysed reach statistical significance in the multivariate analysis, with 12 months of depression as the dependent variable.

## Discussion

To the best of our knowledge, this is the first study to examine the prevalence and socio-demographic and clinical characteristics of FEP with and without suicidal behaviour in a Portuguese population. The present study examined the evolution of the prevalence of suicidal behaviour in the 12 months after FEP in a cohort of Portuguese young people. We also aimed to identify early predictors of suicide behaviour at 12 months of follow-up (baseline and 12 months). In our present study, the prevalence of suicidal behaviour was 25.4% soon after FEP. This is in line with existing studies which showed that approximately 25% to 50% of FEP patients reported suicidal behaviour at initial presentation [7, 20, 31, 63]. We also confirmed that a history of suicidal behaviour before service entry in FEP patients was a predictor of suicidal behaviour at baseline and follow-up. These results are consistent with previous first-episode studies from other countries [47, 64, 65].

As previous published studies showed, we also confirmed that depression was associated with suicidal behaviour [64–67]. Our results showed that depression was a predictor of suicidal behaviour at baseline. Using the univariate analysis, baseline depression was also almost significant as predictor of suicidal behaviour at follow-up (*p* = 0.07). This result suggests that close evaluation, monitoring and early intervention for depression in FEP are crucial to reducing suicidal behaviour in the early phases of psychosis.

Like our study, a majority of the literature did not find an association between negative symptoms and suicidal behaviour [15, 68]. Some studies postulated that patients with significant negative symptoms, namely deficits in emotion expressivity, are impaired from expressing emotional distress caused by psychosis, consequently reducing the probability of developing depression, hopelessness and suicidal behaviour [69]. In line with this, a few studies found negative symptom severity to be inversely related to the risk of suicidal ideation and suicide attempts [65, 67].

Regarding positive symptoms, we failed to demonstrate that these symptoms were predictors of suicidal behaviour, contrary to some prior FEP studies [14, 26]. Our results were similar to those found in other published studies where no predictability was found [47].

An interesting finding of our study is that high levels of total cholesterol were a baseline protector of suicidal behaviour at follow-up. This is in concordance with research in this field of knowledge. Published studies seem to demonstrate an association between low cholesterol and an increased risk of suicide in non-psychotic patients as well as in psychosis [70–74]. There is a dearth of research on the relationship between cholesterol levels and suicidality in FEP. One study found that serum cholesterol concentrations were significantly lower in suicidal than in non-suicidal patients in FEP, suggesting that lower concentrations of serum cholesterol in patients with FEP might be useful as a biological marker of suicidality [70]. Another study in early psychosis demonstrated that lower levels of cholesterol in patients of psychosis were associated with severe suicidal thoughts [72]. The exact mechanisms of the relationship between peripheral cholesterol, brain metabolism and suicidal behaviour are not entirely known. Some authors have suggested an existing abnormality in leptin and lipid metabolism in suicidal behaviour [75]. Moreover, post-mortem brain studies have indicated that violent suicide completers have lower grey-matter cholesterol content, specifically in the frontal cortex [76]. We only included total cholesterol because this metabolic parameter is the only one with previous studies showing association with suicidal behaviour.

Our findings also indicate that a baseline affective psychosis diagnosis and a high score on the PANSS general subscale were potential predictors of baseline suicidal behaviour; however, these results did not reach significant statistical significance in the multivariate analysis. Affective psychosis, which includes major depressive disorder with psychotic symptoms, is a diagnosis associated with suicidal behaviour. The low proportion of patients included with affective psychosis (17.8%) could explain the non-statistical significance of this diagnosis in the multivariate analysis. The PANSS general subscale was composed of 16 items evaluating other symptoms, including depressive symptoms. We argue that these items conditioned the result obtained.

The present study also showed the high frequency of depression and depressive symptoms in the first year after FEP. Almost half of the patients were depressed at baseline, while one-fifth had depression at one year of follow-up. As indicated in the BDI score, the depressive symptoms significantly decreased during the first year after FEP. This is also similar to other published studies [31, 77–79]. Some authors have speculated several pathways to depression in psychosis, namely in schizophrenia. Three possible explanations exist: (1) depression as an intrinsic aspect of the psychotic disorder; (2) depression as a result of the psychotic illness; (3) depression as a result of disturbed developmental pathways [80].

Our findings did not confirm the idea that depressive symptoms in schizophrenia are frequently misdiagnosed with negative symptoms [81]. In this case, high levels of depressive symptoms would exist simultaneously with high levels of negative symptoms, and a significant association between negative and depressive symptoms would have been expected, which did not happen in our study.

The only predictors that we found to be significant to depression at baseline were baseline suicidal behaviour in the last month and a history of suicidal behaviour. Surprisingly, we found no significant baseline predictors of depression at 12 months. Contrary to our findings, other studies found that a long DUP and depressive symptoms at baseline predict depressive symptoms at 12 months of follow-up [77, 82]. Also, some studies found an association between depression scores and positive symptoms and an improvement with successful antipsychotic therapy [39, 83]. However, some studies also failed to find this association, indicating that depressive symptoms may also emerge independently of positive symptoms [84, 85]. The reasons for our study’s failure in finding significant baseline predictors at the 12-month follow-up are not immediately apparent. We could speculate that our baseline evaluation, which was conducted only after a clinical stabilisation of FEP, might have been a little late for significant findings.

Several clinical implications can be drawn from our results. First, at service entry, a significant proportion of patients had a history of suicidal behaviour and suicidal behaviour in the last month. Thus, at the intake of early intervention teams, all patients should undergo a suicide risk assessment. For those at high risk or experience of previous suicidal behaviour, more intensive monitoring and intervention should be offered. Second, given that baseline depression was found to be a predictor of suicidal behaviour, routine assessments for depression should be offered in early intervention teams, and for those with positive screening, close monitoring of the emergence of suicidal behaviour and adequate treatment should be offered. Third, our results also indicate that an evaluation of total cholesterol should be included in the baseline evaluation of FEP patients, not only for the metabolic evaluation but also as a potential predictor of suicidal behaviour. Therefore, patients with low cholesterol should be screened and closely monitoring for the existence and emergence of suicidal behaviour.

The results of this study should be interpreted with some methodological limitations in mind. First, the evaluation of suicidal behaviour (including suicidal ideation, suicide plan and suicide attempt) was mainly obtained from the participants’ self-reports and, thus, was subject to the problem of under-reporting. Second, standardised classification algorithms for suicidal intent, attempts and completed suicides were not used in this research. These algorithms were found to enhance the reliability of the suicidal behaviour assessment. Third, the relatively high proportion of follow-up losses from 118 participants at baseline to 60 at the 12-month follow-up could have limited the results by reducing in some extension the statistical power of the results. Fourth, we studied the predictors of suicidal behaviour, including suicidal ideation, plans and attempts, as a whole group. It was hypothesised that predictors of suicidal ideation, suicide plans and attempts might differ. Fifth, the low numbers obtained regarding suicidal behaviour during follow-up, namely no completed suicide and only 13.3% of participants reporting suicidal behaviour at the 12-month follow-up evaluation. These numbers compromised the statistical power of the study, which could explain the non-significant associations with sex, age of FEP and DUP in our study. Sixth, other important variables that might be potential predictors of suicidal behaviour were not studied because of the difficulties involved in obtaining them, including childhood abuse or previous trauma.

## Conclusions

Our results showed that a large proportion of FEP patients had a history of suicidal behaviour or had current baseline suicidal behaviour. We also verified that depression was prevalent in the 12 months after FEP. Our results are in line with those of previous studies, confirming that a history of suicidal behaviour and depression soon after FEP are associated with suicidal behaviour in the early months after FEP. These findings are important for the development of early intervention programmes to lower the risk of suicide and depression in FEP patients. Our finding regarding the predictive role of baseline total cholesterol should be investigated in further prospective research to determine whether our result was predictive of suicidal behaviour.

## Supplementary Information

**Additional file1:** **Table S1.** Comparison of socio-demographic and clinical characteristics between affective psychosis and schizophrenia spectrum disorders diagnoses.

## Data Availability

Research data can be requested from ricardomcoentre@gmail.com.

## Abbreviations

BDI: Beck Depression Inventory
CI: Confidence interval
DUP: Duration of untreated psychosis
FEP: First-episode psychosis
OR: Odds ratio
PANSS: Positive and Negative Syndrome Scale
GAF: Global Assessment of Functioning
MARS: Medication Adherence Rating Scale
SD: Standard deviation
