# Supplementary material for: Suicidal behaviour after first-episode psychosis: results from a 1-year longitudinal study in Portugal
Source: Ann Gen Psychiatry. 2021 Jul 6;20:35. doi: 10.1186/s12991-021-00356-0 (PMC8262034; doi:10.1186/s12991-021-00356-0)
Supplement: Supplementary file 1 — Additional file1: Table S1. Comparison of socio-demographic and clinical characteristics between affective psychosis and schizophrenia spectrum disorders diagnoses. [file 12991_2021_356_MOESM1_ESM.docx]

|  | Affective psychosis  (n=21) | Schizophrenia spectrum disorders  (n=58) | p-value |
| --- | --- | --- | --- |
| Age mean (SD) | 31.52 (8.66) | 25.50 (6.66) | 0.005 |
| Gender female/male n (%) | 94(42.9)/12(57.1) | 8(13.8)/50(86.2) | 0.005 |
| Education mean (SD) | 12.00 (3.59) | 10.81 (2.98) | 0.078 |
| DUP median (SD) | 34.00 (138.55) | 172.00 (859.13) | 0.007 |
| Cannabis use n (%) | 12 (57.14) | 36 (62.0) | 0.692 |
| PANSS positive mean (SD) | 19.05 (8.63) | 21.55 (7.14) | 0.201 |
| PANSS negative mean (SD) | 16.29 (7.82) | 17.67 (6.95) | 0.365 |
| PANSS general mean (SD) | 39.62 (11.28) | 35.81 (7.47) | 0.345 |
| GAF mean (SD) | 46.90 (17.50) | 40.88 (18.68) | 0.194 |
| BDI mean (SD) | 14.67 (8.21) | 12.24 (10.60) | 0.160 |
| Depression n (%) | 12 (57.1) | 22 (37.9) | 0.143 |
| Suicidal behaviour baseline n (%) | 9 (42.9) | 11 (18.9) | 0.031 |
| History suicidal behaviour n (%) | 11 (52.4) | 18 (31.03) | 0.080 |

**Table** Comparison of socio-demographic and clinical characteristics between affective psychosis and schizophrenia spectrum disorders diagnoses

Abbreviations: DUP = Duration of untreated psychosis; PANSS = Positive and Negative Syndrome Scale; GAF = Global Assessment of Functioning; BDI = Beck Depression Inventory; SD = standard deviation.

Schizophrenia-spectrum disorder included schizophrenia, schizophreniform disorder, delusional disorder and schizoaffective disorder

Affective psychosis included bipolar disorder with psychotic symptoms and depressive disorder with psychotic symptoms
